# High Protein Oral Nutritional Supplements Enable the Majority of Cancer Patients to Meet Protein Intake Recommendations during Systemic Anti-Cancer Treatment: A Randomised Controlled Parallel-Group Study

**DOI:** 10.3390/nu15245030

**Published:** 2023-12-07

**Authors:** Anne-Marie Dingemans, Nico van Walree, Franz Schramel, Magdolen Youssef-El Soud, Edita Baltruškevičienė, Willem Lybaert, Margriet Veldhorst, Claudia. A. van den Berg, Stein Kaasa

**Affiliations:** 1 Department of Pulmonology, Erasmus Medical Center Cancer Institute, 3015 CN Rotterdam, The Netherlands; 2 Department of Pulmonary Diseases, Amphia Hospital, 4818 CK Breda, The Netherlands; nwalree@amphia.nl; 3 Department of Pulmonary Diseases, St. Antonius Hospital, 3435 CM Nieuwegein, The Netherlands; f.schramel@antoniusziekenhuis.nl; 4 Department of Pulmonary Diseases, Máxima Medisch Centrum, 5504 DB Veldhoven, The Netherlands; m.youssef@mmc.nl; 5 Department of Medical Oncology, National Cancer Institute, 08406 Vilnius, Lithuania; edita.baltruskeviciene@nvi.lt; 6 Department of Medical Oncology, VITAZ, 9100 Sint-Niklaas, Belgium; willem.lybaert@telenet.be; 7 Nutricia Research, 3584 CT Utrecht, The Netherlands; margriet.veldhorst@danone.com (M.V.); claudia.van-den-berg@danone.com (C.A.v.d.B.); 8 Department of Oncology, Oslo University Hospital, 0379 Oslo, Norway; stein.kaasa@medisin.uio.no

**Keywords:** oral nutritional supplements, high protein, nutrition support, cancer, malnutrition

## Abstract

ESPEN guidelines recommend a minimum protein intake of 1.0 g/kg body weight (BW) per day to maintain or restore lean body mass in patients with cancer. During anti-cancer treatment, optimal protein intake is difficult to achieve. We investigated whether a high-protein, low-volume oral nutritional supplement (ONS) supports patients in meeting recommendations. A multi-centre, randomised, controlled, open-label, parallel-group study was carried out in nine hospitals (five countries) between January 2019 and July 2021 in colorectal and lung cancer patients undergoing first-line systemic treatment with chemo(radio-) or immunotherapy. Subjects were randomised (2:1) to receive Fortimel Compact Protein^®^ or standard care. Protein intake was assessed with a 3-day food diary (primary outcome). BW was a secondary outcome. Due to challenges in recruitment, the study was terminated prematurely with 42 patients randomised (intervention group (IG) 28; control group (CG) 14). At T1 and T2, protein intake was statistically significantly higher in the IG compared to the CG (1.40 vs. 1.07 g/kg/day at T1, *p* = 0.008; 1.32 vs. 0.94 g/kg/day at T2, *p* = 0.002). At baseline, only 65% (IG) and 45% (CG) of patients met ESPEN minimum protein intake recommendations. However, at T1 and T2 in the IG, a higher proportion of patients met recommendations than in the CG (88% vs. 55% and 40%). No statistically significant difference between study groups was observed for BW. Mean compliance to the ONS was 73.4%. A high-protein, low-volume ONS consumed twice daily enables the majority of patients to reach minimal ESPEN protein recommendations.

## 1. Introduction

It is well established that malnutrition is a common feature not just at diagnosis but throughout the patient’s cancer journey and is associated with treatment toxicity, complications, reduced physical function and reduced survival [1,2]. Furthermore, weight loss is a frequent presenting symptom of a cancer diagnosis with 64% of patients experiencing some degree of weight loss at their first medical oncology visit [3].

Malnutrition prevalence varies by cancer type with the highest frequency of malnutrition seen in gastroesophageal, pancreatic, head and neck, and lung tumours at diagnosis [3]. However, malnutrition is often compounded by metabolic derangements and inflammation induced by the tumour and/or the effects of anti-cancer treatment during the patient’s cancer journey [4]. The inflammatory response can cause anorexia and muscle breakdown that can, in turn, result in a significant loss of body weight, alterations in body composition, and a reduction in physical function [4]. Fortunately, the medical and surgical treatment of all types of cancers is improving with greater sophistication and targeting of individual cancer types. Despite this, malnutrition in cancer remains under-recognised and under-treated with studies showing that only 33–58% of cancer patients at risk of malnutrition actually receive nutritional support [5,6].

Expert guidelines have been published over recent years highlighting the importance of the early recognition and management of the risk of malnutrition throughout the patient’s cancer journey [4,7,8], along with specific guidance on the types of nutritional support, amount and types of nutrients required and their mode of delivery. The role of early nutritional intervention has been further highlighted by the concept of prehabilitation for cancer patients [9], which is increasingly being used in clinical practice for example prior to surgical treatment. Nutritional intervention would enable patients to maintain or strengthen physiological reserves including skeletal muscle to best prepare for an aggressive anti-cancer treatment plan.

An adequate protein intake is one aspect of nutritional intervention to optimise muscle protein anabolism with current cancer specific guidelines from the European Society for Clinical Nutrition and Metabolism (ESPEN) recommending a protein intake of 1.0–1.5 g/kg BW/day [4]. This focus on protein to optimise muscle protein anabolism is also supported by studies showing that the development of sarcopenia in cancer patients is associated with a greater incidence of complications after surgery [10,11], increased treatment toxicity [12,13], and decreased survival [14,15]. This suggests that strategies to reduce the risk of sarcopenia may be beneficial to clinical outcome. Furthermore, lower protein intakes have been associated with muscle wasting during cancer treatment [16,17] and have been shown to be an independent poor prognostic factor in patients with unresectable pancreatic cancer [18].

Studies have shown that optimal protein intake is hard to achieve in practice with 66% of patients with advanced cancer [19] and 52% of head and neck cancer patients [20] receiving systemic anti-cancer therapy failing to meet the ESPEN minimum guidelines for protein intake [4]. Cancer patients undergoing systemic anti-cancer therapy experience a range of nutrition impact symptoms that lead to reduced food intake during their treatment with a concomitant reduction in nutritional status [21]. Dietary choices of cancer patients following diagnosis may be sub-optimal, aligning more with cancer prevention guidelines which encourage higher fruit and vegetable and lower red and processed meat intake with the potential to lower overall protein intake [22]. The European Society of Medical Oncology (ESMO) has recognised the importance of anticipating whether planned anti-cancer treatment is likely to lead to high risk of nutritional decline and provides guidance on adopting a preventive or prophylactic supportive approach to nutritional assessment and individualised intervention with a minimum protein intake of 1.2 g/kg BW/day recommended for adult patients with cancer cachexia [7]. This minimum level is higher than that recommended by ESPEN, but in contrast, the target population of the ESPEN guidelines is all adult cancer patients and cancer survivors independent of severity or stage of disease.

Oral nutritional supplements (ONS) are an established part of the broader nutritional support strategy for cancer patients at risk of malnutrition. Studies have shown that compared to controls who receive dietary advice alone, ONS and dietary advice for 3 months post-surgical discharge result in higher skeletal muscle index, lower sarcopenia prevalence and less chemotherapy modifications (reduced dose, delay or termination) in patients at nutritional risk with colorectal cancer [23] and less weight loss, a higher Body Mass Index (BMI), a higher skeletal muscle index, fewer chemotherapy modifications, less fatigue and less appetite loss in patients at nutritional risk with gastric cancer [24]. High protein intake in hospitalised cancer patients has been shown to improve functional outcomes and quality of life as well as reduce mortality [25]. ONS with a high protein content are of particular interest. In non-malnourished patients with either primary or secondary gastrointestinal or abdominal cavity malignancy, pre-operative high-protein ONS help maintain nutritional status and reduce the number and severity of post-operative complications compared to patients without nutritional support [26]. In colorectal cancer patients, peri-operative high-protein ONS reduce post-operative complications regardless of initial nutritional status, result in a lower risk of rehospitalisation and reduce the cost of treatment during hospitalisation and at six months after surgery compared to routine care [27]. High-protein ONS improve nutritional status (as measured by Subjective Global Assessment, Visual Analogue Scale for appetite, albumin and prealbumin) in pre-cachectic but predominantly malnourished colorectal cancer patients undergoing chemotherapy compared to controls [28] and improve muscle mass and body composition in women with breast cancer undergoing chemotherapy [29].

As part of a preventative approach to limit deterioration in nutritional intake and status, it could be reasonable to attempt to overcome the deficit in protein intake by early intervention using a high-protein, high-energy, low-volume ONS. The current study was undertaken to address a gap in the evidence to ascertain whether a high-protein, high-energy, low-volume ONS could be used to increase protein intake and achieve an adequate protein intake (in line with ESPEN recommendations) in patients with cancer receiving systemic anti-cancer treatment compared to standard care. The study outcomes may be useful to support the implementation of current clinical practice guidelines, which highlight the importance of early nutritional intervention for patients with cancer.

## 2. Materials and Methods

### 2.1. Study Design and Population

This randomised (2:1), controlled, open-label, parallel-group, multi-centre and multi-country study was carried out across five countries (Belgium, Estonia, Lithuania, the Netherlands and Norway) in nine hospital sites.

The study population comprised subjects (age ≥ 18 years) with histologically proven colorectal cancer (CRC) stage IIB, III or IV or histologically or cytologically proven non-small cell lung cancer (NSCLC) stage III or IV and who were scheduled for first-line chemotherapy, concurrent chemoradiotherapy or immunotherapy treatment with a planned duration of at least 12 weeks. Subjects were also required to have a performance status Eastern Cooperative Oncology Group (ECOG) score of either 0 or 1 and be able to provide written informed consent to take part in the study. Exclusion criteria included weight loss of >10% in the last 6 months and BMI < 20.0 kg/m^2^. Full details of inclusion and exclusion criteria are given in Table S1.

The study was conducted according to the International Council for Harmonisation of Technical Requirements for Pharmaceuticals for Human Use (ICH) Good Clinical Practice (GCP) principles and in compliance with the ‘World Medical Association Declaration of Helsinki’ (64th WMA General Assembly, Fortaleza, Brazil, October 2013) and with the local laws and regulations of the countries where the study was performed. The protocol was submitted to the local ethics committees and approved prior to the start of the study. The study was registered in the www.clinicaltrials.gov database with identifier NCT05677958 and reported according to the CONSORT statement [30].

This study was affected by the coronavirus disease (COVID-19) outbreak, which meant that recruitment stopped in March 2020 and restarted 2–5 months later depending on the study site.

### 2.2. Nutritional Intervention

Subjects were randomly allocated to either the intervention group (IG) or control group (CG). The IG received, twice daily, a low-volume (125 mL) high-protein (18 g protein), energy-dense (300 kcal) ONS (Fortimel/Nutridrink Compact Protein^®^, Nutricia, commercial name varied by country) in addition to normal oral intake. Subjects were offered a variety of flavours, one serving to be taken in the morning and one in the afternoon or evening, and were instructed to start taking the study product within seven days before the start of the first anti-cancer treatment cycle. The total intervention period ended 12 weeks after initiation of their first-line anti-cancer treatment. The CG received standard care allowing for any type of nutritional support according to hospital standard practice. Equally, if it was deemed necessary, as a result of clinician’s assessment, subjects in the IG as well as in the CG could receive additional servings of the ONS to fully meet their nutritional requirements.

### 2.3. Randomisation Procedure

The randomisation sequence was generated using the PLAN procedure of SAS statistical software (Enterprise Guide version 4.3 or higher). The permuted block randomisation was stratified for cancer type and treatment (chemotherapy, concurrent chemoradiotherapy or immunotherapy). The allocation ratio was 2:1 for the IG and the CG, respectively. An independent statistician generated the allocation sequence and decided the block sizes, which were random. Details about sequence generation, block size or sizes and whether the block size(s) were fixed or random were unknown to the investigator and research site staff who were responsible for enrolling eligible subjects into the study and assigning them to their given randomisation numbers.

### 2.4. Outcome Measures

As subjects had anti-cancer treatment regimens with treatment cycles of varying length (2, 3 or 6 weeks); assessment timepoints differed accordingly. Assessments at timepoint 0 (T0) were related to the start of the study (baseline); assessments at timepoint 1 (T1) were related to the planned end of the first treatment cycle, i.e., assessments took place at the end of week 2, 3 or 6 according to the planned duration of a treatment cycle; assessments at timepoint 2 (T2) were related to the planned end of treatment cycle 2 in case of treatment schedules with 3-week treatment cycles or to the planned end of treatment cycle 3 in case of treatment schedules with 2-week cycles. Assessments at timepoint 3 (T3) occurred at the end of the 12-week intervention period for all subjects. A treatment cycle was defined as the interval between the start of administration of each treatment sequence and the next.

The primary outcome measure was defined as the average protein intake per day (g/day; g/kg BW/day) corrected for baseline (T0) after one cycle of first-line anti-cancer treatment assessed with a three-day food diary completed at T0 and at T1. The secondary outcome measures included the proportion of subjects with a protein intake ≥1.0 g/kg BW/day, which represents the lower limit of the ESPEN recommendations for protein intake for cancer patients, at T1 and also a change in body weight during the 12-week study period.

Food diaries were completed by subjects at home and validated by study staff at the next hospital visit. Analysis of food diaries was undertaken by a local dietitian using country-specific nutritional analysis software. Compliance was monitored in the IG using a diary where the subject documented the amount of study product consumed (0–¼–½–¾–1) per serving. Any reasons for non-compliance were also recorded. Body weight was measured using a calibrated weighing scale at all timepoints (T0-3).

**Figure 1:**
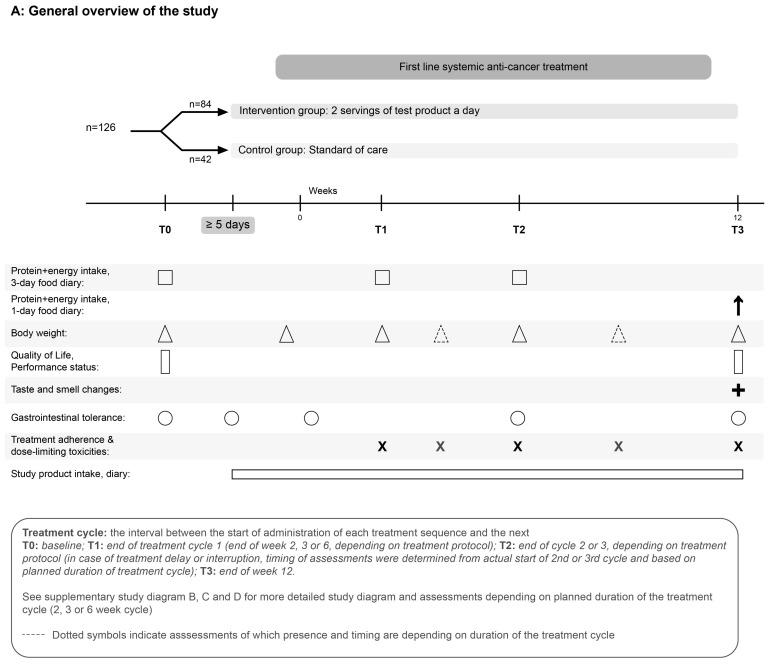
Schematic representation of the study design. Timing of some measurements vary depending on the length of the anti-cancer treatment cycle (see Supplementary Material Figure S1B–D).

Other exploratory outcome measures were included: average protein intake per day (g/day; g/kg BW/day) corrected for baseline at T2 and T3, total dietary energy intake (kcal/kg BW/day) and the proportion of subjects with a protein intake ≥1.0 g/kg BW/day at T0 and at T2 and T3. Factors impacting dietary intake were also included as exploratory outcome measures: symptoms that impacted dietary intake and alteration in taste and smell perception questionnaire. Health-related quality of life was assessed using the European Organisation for Research and Treatment of Cancer Quality of Life Questionnaire-Core 30 (EORTC QLQ-C30) [31,32] at T0 and at T3. Functional status was assessed using the ECOG score [33] at T0 and at T3. Anti-cancer treatment adherence and dose-limiting toxicities were recorded. A general overview of the study timeline and timing of measurements is shown in Figure 1. Further details of the methodology and timelines according to the subjects’ anti-cancer treatment cycle are given in Supplementary Materials.

Adverse events (AEs) were recorded using the National Cancer Institute Common Terminology Criteria for Adverse Events (NCI CTCAE) Version 4.0 system [34]. A gastrointestinal (GI) tolerance questionnaire was also administered to subjects at T0 and at specified intervals during the study.

### 2.5. Statistics and Data Analysis

Sample size calculation was based on estimates from previous studies on the effect of nutritional intervention on protein intake in cancer patients with an expected mean difference in protein intake of 16 g in the IG vs. the CG (standard deviation (SD) of difference: 24 g). Based on these estimates and using a significance level (α) of 0.05, power of 80% and a 2:1 randomisation, a sample size of 84 subjects (56 IG and 28 CG) was calculated based on a two-sample *t*-test. This was assumed to be sufficient to detect a statistically significant difference in protein intake at the end of the first treatment cycle between the IG and the CG. A 2:1 randomisation was chosen to offer subjects a greater chance of receiving the nutritional intervention to incentivise participation in the study. Anticipating a drop-out rate of 33% based on previous comparable studies [35,36], the total number of subjects to be included in the study was 126 (84 IG and 42 CG).

All analyses were performed with the modified Intention-To-Treat (mITT) subject data set, i.e., subjects who had completed the first treatment cycle (primary endpoint for protein intake). Descriptive statistics were reported as mean (SD) and/or median (interquartile range (IQR)) for skewed distributed data, or as *n* (%).

Inferential statistics were only performed for the primary outcome parameter using an Analysis of Covariance (ANCOVA) model with T1 values as outcome and T0 values and the group as covariates, and adjusting for cancer type and cancer treatment as stratification factors. The normality assumption was checked and satisfied for ANCOVA analysis, so the model provided a good fit despite the small sample size. Furthermore, t-tests and the Wilcoxon rank-sum test were performed, and only the *t*-test *p*-values were provided for all the other timepoints and for other parameters, as there was no difference in the conclusion between parametric and non-parametric tests.

All analyses were performed with SAS version 9.4 (SAS Institute Inc., Cary, NC, USA) [37,38].

## 3. Results

### 3.1. Patient Characteristics

The study was conducted between January 2019 and July 2021. Due to recruitment difficulties, the study was terminated early.

**Figure 2:**
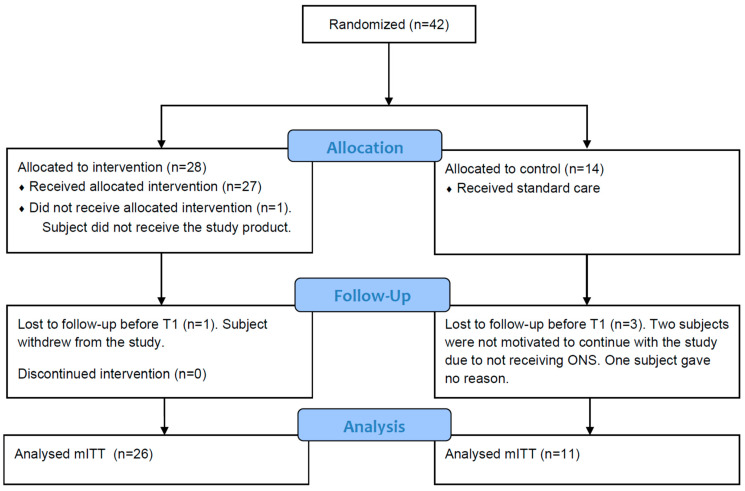
Flow diagram for selection, randomisation and analysis of study subjects [30] (mITT, modified Intention-To-Treat).

Figure 2 shows the flow of subjects throughout the study for all study groups. A total of 44 subjects were enrolled of which 42 subjects were randomised; 37 subjects (26 in the IG and 11 in the CG) were included in the primary analysis (mITT) and 29 subjects completed the study.

The main demographic data and subject characteristics at baseline are summarised in Table 1. The mean (SD) age of the subjects was 66.1 (7.8) years in the IG and 70.1 (8.2) years in the CG. There were more female subjects in the IG compared to the CG (57.7% vs. 27.3%). Regarding primary tumour localisation, there were more subjects with NSCLC cancer in the CG (72.7%) compared to the IG (53.8%). In both groups, approximately 30% of subjects had previous cancer treatment in the past 12 months. The majority of subjects had tumour stage IV at diagnosis (IG: 73.1%, CG: 80.0%) and underwent chemotherapy during the intervention period (IG: 73.1%, CG: 63.6%). Subjects in both groups were relatively well nourished at baseline with a mean (SD) BMI in the overweight category (IG: 26.2 (3.7) kg/m^2^ and CG: 27.2 (3.0) kg/m^2^) and mean (SD) weight loss in the previous 6 months of <5% (IG: 3.9 (2.8)% and CG: 4.7 (2.5)%). At baseline, only 65% and 45% of subjects in the IG and CG, respectively, met ESPEN minimum protein intake recommendations [4]. Full details of anthropometric and nutritional parameters at baseline are shown in Table 2.

### 3.2. Protein Intake

**Figure 3:**
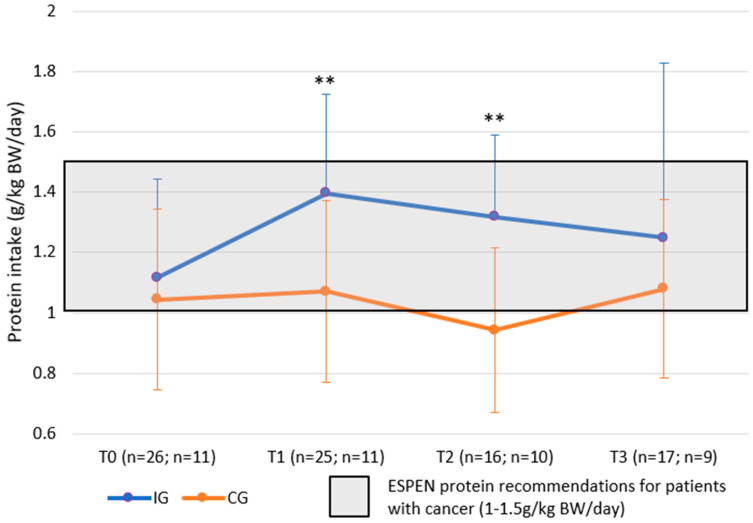
Protein intake g/kg BW/day (mITT). ESPEN recommendation for protein intake: 1.0–1.5 g/kg BW/day; mITT, modified Intention-to-Treat; BW, body weight ** *p* < 0.01.

Mean BW adjusted protein intake was statistically significantly higher in the IG compared to the CG at both T1 (primary endpoint) and T2 (IG: 1.40 vs. CG: 1.07 g/kg BW/day at T1 *p* = 0.008 based on *t*-test; IG: 1.32 vs. CG: 0.94 g/kg BW/day at T2, *p* = 0.002 based on *t*-test) (Table 3 and Figure 3). There was no difference between groups at T3.

Estimated mean protein intake was statistically significantly higher in the IG vs. CG at T1: IG 106.1 g/day (95% CI: 95.2–117.0 g/day) vs. CG 88.8 g/day (95% CI: 74.2–103.4 g/day) with a treatment difference of 17.2 g/day (95% CI: 3.0–31.5 g/day, *p* = 0.019 based on ANCOVA) (Table 4). Similarly, the estimated mean BW adjusted protein intake values at T1 were 1.40 g/kg BW/day (95% CI: 1.24–1.56 g/kg BW/day) for the IG vs. 1.15 g/kg BW/day (95% CI: 0.94–1.36 g/kg BW/day) for the CG with a treatment difference of 0.25 g/kg BW/day (95% CI: 0.05–0.46 g/kg BW/day, *p* = 0.018 based on ANCOVA test); see Table 4.

The change in protein intake from T0 to T3 (g/kg BW/day) in both groups was not statistically significant.

### 3.3. Proportion of Subjects with a Protein Intake ≥1.0 g/kg BW/day

**Figure 4:**
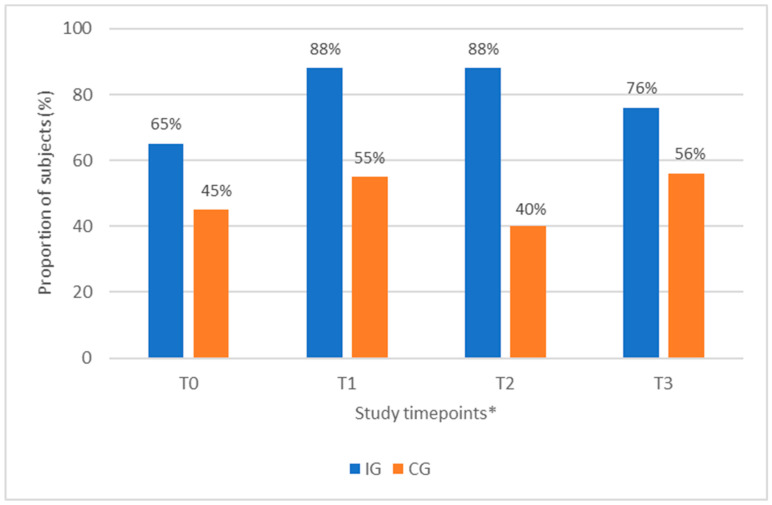
Proportion of subjects (%) with protein intake ≥1.0 g/kg BW/day at study timepoints T0, T1, T2, T3 in IG and CG (mITT). * At all timepoints, there was some missing data: at T1 IG: *n* = 1; T2 IG: *n* = 10, CG: *n* = 1; T3 IG *n* = 9, CG: *n* = 2.

At baseline, 65% of the IG had a protein intake of ≥1.0 g/kg BW/day, i.e., above the lower limit of the ESPEN recommendation for protein intake for adult cancer patients. This increased to 88% at T1 and at T2, and it was 76% at T3. For the CG, the proportion of subjects who had a protein intake of ≥1.0 g/kg BW/day was 45% at baseline, and this increased to 55% at T1; see Figure 4 (Table S2).

### 3.4. Change in Body Weight during 12 Weeks of Anti-Cancer Treatment

The mean body weight in the IG increased by 0.55 kg from T0 to T1 and by 0.74 kg from T0 to T2. For the CG, mean body weight decreased by −0.09 kg from T0 to T1 and by −0.46 kg from T0 to T2. At T3, both groups showed an increase in mean body weight of 0.81 kg and 0.84 kg compared to T0, respectively, for the IG and CG (Table S3). No statistically significant differences in body weight were observed between the IG and CG at any of the timepoints.

### 3.5. ONS Compliance

**Figure 5:**
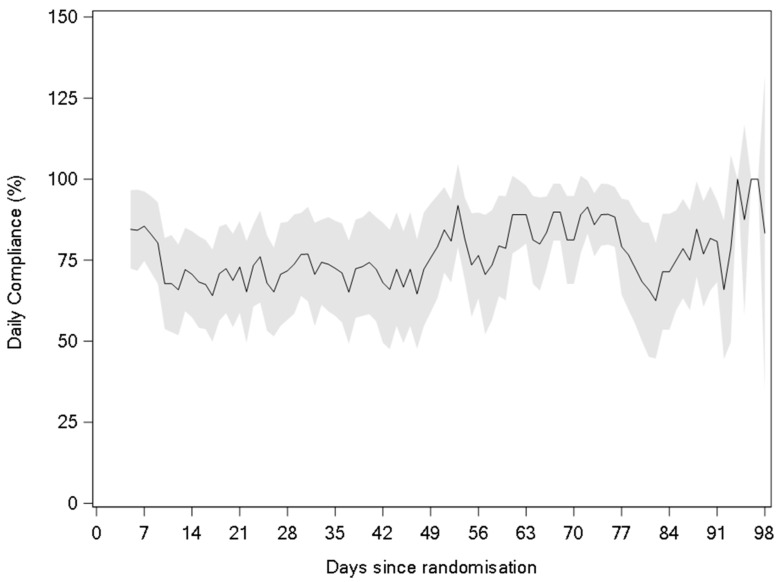
Study product compliance during the 12-week intervention (mean with 95% CI) (mITT).

Patient compliance with the ONS prescription was measured in the IG with a mean ± SD compliance during the study of 73.4% ± 23.8%. This approximately equates to 1.5 servings of the study product per day. More than 50% of the subjects in the IG had a study product compliance of ≥80% during the intervention. Figure 5 shows the study product compliance over time. The most frequently reported reasons for not consuming the full serving of the ONS were feeling full/satiety (54%), no appetite (46%), nausea or vomiting (42%), and fatigue (35%).

### 3.6. Gastrointestinal (GI) Tolerance and Safety

Subjects in the IG reported more adverse events (AEs) compared to the CG 66.7% vs. 28.6%, respectively, and the difference was statistically significant (*p* = 0.022, 95% CI =38.1% (5.5%, 62.7%)). The most frequently reported AEs were GI related (nausea: IG 11.1% (*n* = 3), CG 0.0% (*n* = 0); and diarrhoea: IG 7.4% (*n* = 2), CG 0.0% (*n* = 0)) and with a mild severity (e.g., grade 1 or 2) as expected in a nutritional intervention. Serious adverse events (SAEs) were only experienced in the IG, and these were not related to study product intake but were mainly related to infections (e.g., coronavirus infection) or general disorders and administration of site condition.

The medical history and concomitant medications of the participating subjects were representative of the cancer population. None of the subjects presented with any of the prohibited ailments at the inclusion in the study. Based on the available data, no major health concerns were observed in this study.

### 3.7. Exploratory Outcome Measures

The descriptive results of the key exploratory outcome measures are described here with the remainder described in the Supplementary Material.

The median (Q1–Q3) of the Global Quality of Life/Global Health Status score was lower for the IG than for the CG at T0 (IG: 66.67 (58.33–83.33) vs. CG: 83.33 (50.00–91.67)). However, at T3, the IG had the same median score as they had reported at T0 (66.67 (50.00–83.33)), while the CG reported a lower score compared to their T0 score (66.67 (58.33–83.33)).

A proportion of subjects, in both groups, experienced at least one treatment dose reduction of >10% (IG: 26.9% vs. CG 36.4%). Reported dose reductions were mainly due to haematological toxicity or non-treatment-related reasons. Similarly, a proportion of subjects in both groups experienced at least one treatment delay, interruption, or premature stop (IG: 38.5%, CG: 27.3%).

Two subjects in the IG consumed an additional ONS for, respectively, 3 and 8 intervention days. In the CG, only one subject received a prescription of ONS (Fortimel^®^ Compact Protein) for two days. None of the subjects in either the IG or CG received enteral nutritional support during the study.

## 4. Discussion

Despite the challenges in patient recruitment and early termination of this study, we demonstrated that intervention twice daily with a high-protein, high-energy, low-volume ONS was effective at increasing protein intake in patients with CRC and NSCLC undergoing first-line systemic treatment with chemo-, concurrent chemoradio- or immunotherapy. Data from the literature suggest that 52–66% of patients undergoing systemic treatment fail to meet ESPEN minimum protein recommendations of >1.0 g/kg BW/day without specific nutritional support [19,20]. In our study, 55% of the subjects in the CG at baseline and 45% at T1 failed to meet the ESPEN minimum recommended protein intake of 1.0 g/kg BW/day, despite receiving routine nutritional care, which is in line with the estimates in the literature. Conversely, 35% of subjects in the IG at baseline and 12% at T1 failed to meet the minimum recommended protein intake, suggesting that the twice-daily ONS was effective at ensuring the majority of subjects consumed the recommended protein intake. As a low protein intake is reported to be associated with low muscle mass [16,17], cancer-related fatigue [19,39] and poorer overall survival [19], the increase in protein intake achieved in our study is clinically relevant. Our study also showed that the estimated mean protein intake achieved by subjects in the IG after the first cycle of anti-cancer treatment was 1.4 g/kg BW/day, which is towards the upper end of the ESPEN recommendations and above the 1.2 g/kg BW/day minimum recommended intakes by ESMO [7]. A review of the literature suggests that patients with head and neck, lung, and oesophageal cancer who maintain a protein intake above 1.4 g/kg BW/day were more likely to maintain muscle mass during treatment [17].

Body weight changes showed a trend towards better weight maintenance at T1 and T2 in the IG compared to the CG; however, these changes did not reach statistical significance. At T3, both the IG and CG showed an increase in body weight compared to T0. An important factor for consideration in the current study is the nutritional status of subjects at baseline. Anorexia, inadequate nutritional intake and cachexia are highly prevalent in patients with gastrointestinal or lung cancer at diagnosis [3,40]. Within this study, we focused on an early intervention approach in patients without significant weight loss and with mild to moderate malnutrition, according to the ESMO guidelines [7]. This was intentional, as the aim was to provide an ONS early to patients in the IG to take a more preventative approach to weight loss in line with ESMO guidelines. It is surprising that more weight loss was not observed in the CG despite this being well documented in the literature with reports of 68% of lung cancer patients experiencing some degree of weight loss [41] and up to 49% experiencing >5% weight loss in a six-month period [42]. Similar results have been reported in CRC with up to 48% of patients experiencing >5% weight loss over six months [42].

A review of the literature highlighted that muscle loss is also prevalent in these cancer types with a median sarcopenia prevalence of 49% and 70% in CRC and lung cancer patients, respectively [42]. A significant reduction in muscle area of 6.1% has also been reported in patients with metastatic CRC during 3 months of chemotherapy [43]. The clinical significance of weight and muscle loss is well established. Weight loss has been shown to be an independent negative prognostic factor for survival in NSCLC patients treated with chemotherapy [44], and the severity of malnutrition (as graded by the Global Leadership Initiative on Malnutrition (GLIM) criteria has been associated with reduced overall survival [1]. Low muscle mass is also associated with treatment toxicity in advanced NSCLC [45] and colorectal cancer [46,47] and is an independent negative prognostic factor for survival in these cancer types [48,49,50,51]. Whilst our study showed a trend towards better weight maintenance in the IG, other intervention studies with ONS have shown significant improvements in body weight, nutritional status and muscle mass in patients with cancer with a variety of tumour types [28,29,52].

Although no statistically significant increase in body weight was observed, the increase in protein intake seen in the IG may exert a positive influence on body composition with the potential to maintain or increase lean body mass [17]. Previous nutritional intervention studies with high protein ONS have demonstrated significant increases in muscle mass in pancreatic and bile duct cancer patients undergoing systemic anti-cancer treatment [52]. Similarly, among breast cancer patients receiving chemotherapy, a high-protein, high-energy, low-volume ONS, twice daily, improved body composition with a significant increase in muscle mass, fat-free mass and fat-free mass index demonstrated [29]. Further studies should investigate the effect of high-protein, low-volume ONS in CRC and NSCLC patients on body composition, as the link between skeletal muscle depletion and prognosis in cancer patients is well established [53].

The study product had a low volume (125 mL) to support patient compliance throughout the course of the treatment. Overall compliance to the study product was high (73.4%, i.e., 1.5 servings per day with 2 servings being the prescribed dose) and remained stable over the 12-week intervention period. A previous study showed an average ONS compliance of around 50% in patients with stage III NSCLC during multimodality treatment [36], and a 60% compliance rate was seen in head and neck cancer patients undergoing radiotherapy [54], suggesting that the current study has a relatively high rate of compliance.

We observed no major differences in the other outcomes assessed such as quality of life, performance status, factors affecting dietary intake, taste and smell alterations, and treatment tolerance and toxicities between study groups, although the reporting of these parameters was significantly hindered by the small sample size. Data from other studies using ONS for three months, post-discharge, following colorectal cancer surgery have shown improvements in other outcomes such as reduced skeletal muscle loss and sarcopenia prevalence as well as improved chemotherapy tolerance compared with dietary advice alone in patients at nutritional risk [23]. Similar results were found in malnourished gastric cancer patients whereby ONS provided post-operatively for three months improved skeletal muscle maintenance, chemotherapy tolerance and some quality of life variables (fatigue and appetite loss) [24]. Future studies should investigate the effect of early or assertive nutritional intervention from diagnosis on body composition and clinical outcomes along the projected clinical course to establish how meeting nutritional needs early, before overt malnutrition develops, can result in better clinical outcomes.

Despite the challenges in patient recruitment, this study was a well-designed, multi-centre, randomised controlled trial in which 42 patients were enrolled, and subsequent data on nutritional outcomes was successfully collected. The study adds to the existing literature by focusing on a cohort of patients that excluded the most malnourished subjects but represented an “at-risk” group based on their projected clinical course according to guidelines from the ESMO [7]. Alongside the nutritional data collected, ONS compliance was also measured over the entire 12-week study period; this adds strength to the study, as it provides some insight into the application of the intervention in clinical practice.

In contrast to the strengths of this study, there are a few limitations. Recruitment challenges led to the study being terminated earlier than planned with 37 patients being included in the modified Intention-to-Treat (mITT) subject data set (subjects who completed the primary endpoint at T1). The study was also impacted by the COVID-19 pandemic, which meant that recruitment was paused for an extended period of time. This may have affected ongoing recruitment with subjects being less willing to have non-essential healthcare interactions during the pandemic given their clinical vulnerability. A review of the impact of COVID-19 on oncology clinical trials showed that patient enrolment in active clinical trials for cancer therapies was severely affected by the COVID-19 pandemic, and recruitment continued to be problematic in some countries [55]. Furthermore, as an advanced cancer population has been enrolled to this study, a dropout rate between T1 and T2 could be observed mainly due to product-unrelated AEs and withdrawals by subjects.

Despite using a 2:1 randomisation to offer subjects a greater chance of receiving the nutritional intervention, it is possible that the lack of a control product may have affected patients’ willingness to participate and/or continue in the study (contributing to higher attrition in the control arm). A 2:1 randomisation approach is more often used in oncology trials so that patients have a higher chance of receiving the intervention and thus the expected benefit from participation [56,57]. The lack of control product also biased the evaluation of AEs between the IG and CG and made blinding impossible in this study. The literature also shows that the blinding of subjects to different modes of nutritional intervention is, in many cases, very difficult or even impossible [58]. Moreover, the primary endpoint was assessed with a 3-day food diary in a multi-country setting, which may have introduced a number of variables due to differences in data collection, validation and data entry. Lastly, recruitment difficulties resulted in a smaller number of subjects who completed the study. Patients may be offered a choice to participate in other clinical studies in relation to their cancer care, e.g., new treatment modalities or investigational therapies which would preclude enrolment in nutritional intervention studies, thereby limiting the pool of potential subjects. Several patients judged the amount of time required to record their dietary intake as troublesome; future studies could investigate alternative methods such as electronic recording of intake via an app.

A multi-centre international study design was selected for this trial for a number of reasons, namely to maximise recruitment in a population where recruitment can be difficult and to reflect the diverse practices and expertise that occur in real-world clinical practice to increase the generalisability of results. However, this can also represent a challenge since practices such as standard nutritional care can differ between study centres. The study was not stratified by centre due to more important covariates being prioritised, i.e., cancer type and cancer treatment. However, at study initiation a questionnaire about standard nutritional care was completed via interview with the relevant study site personnel. Heterogeneity in when, how and by whom patients were screened for nutritional risk and when and what nutritional intervention was used was identified. The prescription of ONS was found to be part of standard nutritional care but generally commenced relatively late in the patient journey, although this varied by country. For this reason, the use of ONS in the control group in the study was monitored. One subject in the control group received a prescription of ONS (one serving of 125 mL per day) on intervention Day 3 and 4, which is unlikely to have influenced the results.

Despite a growing body of research demonstrating the benefits of nutritional intervention in cancer, there remains a gap in translating the available evidence into clinical practice. The provision of nutritional intervention is still an unmet need for many patients despite the availability of nutritional guidelines from expert professional groups. Research that considers the patients’ perspective shows that nutrition is rated as highly important in their cancer care [59], but nutritional advice is not always consistently available even for patients with identified malnutrition [5]. It is clear that greater effort is required to ensure that nutritional care is offered early to cancer patients and continued throughout their cancer journey. National and local policy changes are required to ensure the adoption into clinical practice of systems and pathways that support all steps in nutritional care from screening, assessment, diagnosis, early and appropriate intervention to ongoing monitoring and evaluation.

## 5. Conclusions

Without specific nutritional intervention, cancer patients often fail to meet the minimum protein intakes recommended by ESPEN guidelines. Adequate protein intake is important to prevent nutritional deterioration and can support muscle mass and function and improve outcomes during treatment. Our results show that the high-protein, high-energy, low-volume ONS was effective at increasing protein intake in line with ESPEN guidelines in a cohort of CRC and NSCLC patients during their first cycle of systemic anti-cancer treatment. Compliance with the ONS was high, suggesting that this could be a nutritional therapy offered early in the patient’s cancer journey that would be feasible to implement into clinical practice. Future research that explores innovative strategies for the recruitment and retention of cancer patients in nutritional studies is warranted.

## Figures and Tables

**Table 1:** Demographic data and subject characteristics at baseline by study group (mITT).

Parameter	IG (*n* = 26)	CG (*n* = 11)	Total (*n* = 37)
Sex, *n* (%)			
Female	15 (57.7%)	3 (27.3%)	18 (48.6%)
Male	11 (42.3%)	8 (72.7%)	19 (51.4%)
Age (years), mean (SD)	66.1 (7.8)	70.1(8.2)	67.3 (8.0)
Smoking, *n* (%)			
No	23 (88.5%)	10 (90.9%)	33 (89.2%)
Yes	3 (11.5%)	1 (9.1%)	4 (10.8%)
Previous cancer treatment in last 12 months, *n* (%)			
No	18 (69.2%)	8 (72.7%)	26 (70.3%)
Yes	8 (30.8%)	3 (27.3%)	11 (29.7%)
Localisation primary tumour, *n* (%)			
Colorectal	12 (46.2%)	3 (27.3%)	15 (40.5%)
Lung	14 (53.8%)	8 (72.7%)	22 (59.5%)
Tumour stage at diagnosis, *n* (%)			
IIB	1 (3.8%)	0 (0.0%)	1 (2.8%)
III	6 (23.1%)	2 (20.0%)	8 (22.2%)
IV	19 (73.1%)	8 (80.0%)	27 (75.0%)
Missing	0	1	1
Anti-cancer treatment during intervention, *n* (%)			
Chemotherapy	19 (73.1%)	7 (63.6%)	26 (70.3%)
Concurrent chemoradiotherapy	2 (7.7%)	1 (9.1%)	3 (8.1%)
Immunotherapy	5 (19.2%)	3 (27.3%)	8 (21.6%)
Planned duration treatment cycle, *n* (%)			
2 weeks	8 (30.8%)	2 (18.2%)	10 (27.0%)
3 weeks	17 (65.4%)	8 (72.7%)	25 (67.6%)
6 weeks	1 (3.8%)	1 (9.1%)	2 (5.4%)

IG, intervention group; CG, control group; SD, standard deviation.

**Table 2:** Subject anthropometric and nutritional characteristics at baseline by study group (mITT).

Parameter	IG (*n* = 26)	CG (*n* = 11)	Total (*n* = 37)
Body weight (kg) mean (SD)	75.2 (10.6)	82.3 (15.9)	77.4 (12.6)
Body mass index (kg/m^2^) mean (SD)	26.2 (3.7)	27.2 (3.0)	26.5 (3.4)
Proportion of patients who experienced unplanned WL *n* (%)	10 (38%)	8 (73%)	18 (49%)
Unplanned WL in last 6 months (kg) mean (SD)	3.0 (2.3) ^a^*	3.9 (2.5) ^b^	3.4 (2.4) ^c^
Unplanned WL in last 6 months (% BW) mean (SD)	3.9 (2.8) ^a^	4.7 (2.5) ^b^	4.3 (2.6) ^c^
Energy intake (kcal/kg BW/day) mean (SD)	28.0 (9.1)	24.5 (7.3)	27.0 (8.6)
Protein intake (g/kg BW/day) mean (SD)	1.12 (0.33)	1.04 (0.30)	1.10 (0.32)
Protein intake ≥ 1.0 g/kg BW/day (lower limit of ESPEN recommendation for protein intake in adult cancer patients) *n* (%)			
Yes	17 (65%)	5 (45%)	n/a
No	9 (35%)	6 (55%)	n/a

mITT, modified Intention-to-Treat; IG, intervention group; CG, control group; SD, standard deviation; WL, weight loss; BW, body weight; ^a^ *n* = 10, ^b^ *n* = 8, ^c^ *n* = 18; * In the IG, there was 1 subject with a missing value for this parameter; n/a, not applicable.

**Table 3:** Protein intake (g/kg BW/day) at T0, T1, T2 and T3 (mITT).

Parameter	Number of Subjects (IG vs. CG)	IG (*n* = 26)	CG (*n* = 11)
Protein intake (g/kg BW/day) mean (SD)			
T0 ^a^	*n* = 26 vs. *n* = 11	1.12 (0.33)	1.04 (0.30)
T1	*n* = 25 vs. *n* = 11	1.40 (0.33)	1.07 (0.30)
T2	*n* = 16 vs. *n* = 10	1.32 (0.27)	0.94 (0.27)
T3	*n* = 17 vs. *n* = 9	1.25 (0.58)	1.08 (0.30)

BW, body weight; mITT, modified Intention-to-Treat; IG, intervention group; CG, control group; SD standard deviation. ^a^ Food diary at T0 was completed before study product intake had started in IG.

**Table 4:** Estimated mean difference in protein intake at T1, g/day and g/kg BW/day (mITT).

Parameter	LS Means ± SE	95% CI	Treatment Difference ± SE	95% CI	*p*-Value ^1^
**Protein intake (g/day)**					
IG	106.1 ± 5.3	95.2–117.0	17.2 ± 7.0	3.0–31.5	0.019 *
CG	88.8 ± 7.2	74.2–103.4			
**Protein intake (g/kg BW/day)**					
IG	1.40 ± 0.08	1.24–1.56	0.25 ± 0.10	0.05–0.46	0.018 *
CG	1.15 ± 0.10	0.94–1.36			

^1^ *p*-value is based on ANCOVA model with T1 values as outcome and T0 values and group as covariates and adjusting for cancer type and cancer treatment as stratification factors. * Statistically significant, *p* ≤ 0.05. BW, body weight; CI, confidence interval; LS, least square; SE, standard error.

## Data Availability

The datasets presented are available upon reasonable request. Any requests will be reviewed against compliance with ethical, scientific, regulatory, and legal requirements. Requests to access the datasets should be directed to claudia.van-den.berg@danone.com.

## Supplementary Materials

The following supporting information can be downloaded at https://www.mdpi.com/article/10.3390/nu15245030/s1. Table S1: Full inclusion and exclusion criteria; Figure S1: Schematic representation of the study design for subjects with a 2-week anti-cancer treatment cycle (B), for subjects with a 3-week anti-cancer treatment cycle (C) and for subjects with a 6-week anti-cancer treatment cycle (D); Table S2: Proportion of subjects with a protein intake above the lower limit of the ESPEN recommendations (≥1.0 g/kg BW/day) for protein intake for cancer patients (mITT); Table S3: Change in body weight during the 12-week study period (mITT).
