# Supplementary material for: High Protein Oral Nutritional Supplements Enable the Majority of Cancer Patients to Meet Protein Intake Recommendations during Systemic Anti-Cancer Treatment: A Randomised Controlled Parallel-Group Study
Source: Nutrients. 2023 Dec 7;15(24):5030. doi: 10.3390/nu15245030 (PMC10745925; doi:10.3390/nu15245030)
Supplement: Supplementary file 1 [file nutrients-15-05030-s001.zip › nutrients-2665152-supplementary.pdf]

SUPPLEMENTARY MATERIAL

Table S1: Full inclusion and exclusion criteria

Inclusion criteria	Exclusion criteria
Histologically proven CRC stage IIB, III or IV or histologically or cytologically proven NSCLC stage III or IV	Scheduled for first line chemotherapy, concurrent chemoradiotherapy or immunotherapy treatment starting ≤4 days after randomization
Eligible and scheduled for first line chemotherapy, concurrent chemoradiotherapy or immunotherapy treatment with a planned duration of at least 12 weeks	Received >10 doses of radiotherapy within 2 months prior to the study
Performance status Eastern Cooperative Oncology Group (ECOG) score 0 or 1	Weight loss >10% in the last 6 months
Age ≥ 18 years	Body Mass Index < 20.0 kg/m^2
Able to provide written informed consent	Life expectancy < 3 months
	Prescription of Oral Nutritional Supplement before start of first line treatment based on hospital's standard practice
	Presence of ileostoma or ileal pouch
	Contra-indications to oral feeding, high protein nutrition or to the test product (including galactosaemia) in the opinion of the investigator
	Known pregnancy or lactation
	Current alcohol or drug abuse in the opinion of the investigator
	Investigator's uncertainty about the willingness or ability of the subject to comply with the protocol requirements
	Participation in any other studies involving investigational or marketed products concomitantly or within two weeks prior to entry into the study

SUPPLEMENTARY MATERIAL

Method – additional information

At the initial baseline assessment (time point 0, T0) subjects' socio-demographic data and medical history were collected. Body mass index (BMI kg/m^2) and unplanned body weight loss in the last six months (kg,%) were calculated. Tumor stage at diagnosis [TNM-stage] and localization of primary tumor were recorded.

Individual subjects' anti-cancer treatment programme was also recorded and included the type of chemotherapy/immunotherapy drug and planned duration of treatment cycles (2 weeks/3 weeks/6 weeks). In the case of concurrent chemoradiotherapy: the planned start of radiotherapy related to the start of chemotherapy (same day/day 1 of cycle 2 etc.) and the planned dosage and fractionation of radiotherapy (dosage in Gray/fraction (Gy/fraction)); total number of fractions per day, number of fractions per week and planned total number of fractions.

Subjects had anti-cancer treatment regimens with treatment cycles with a planned duration of 2, 3 or 6 weeks. In case of a delay or interruption of a treatment cycle, the timing of assessments was determined from the actual start of the second or third cycle and based on the planned duration of the treatment cycle. The actual start of the second or third cycle was considered as a reference to schedule the timing of assessments at T2. A schematic overview of the study giving details of timing of measurements for subjects with a 2, 3 or 6 week anti-cancer treatment cycle are given in Figure S1,B ,C, D respectively.

Subjects completed a 3-day food diary to assess dietary intake at the end of the first treatment cycle, being at the end of week 2, 3 or 6 of the intervention (T1). At the end of the second treatment cycle (in case of treatment protocols with 3 week cycles) or at the end of the third treatment cycle (in case of treatment protocols with 2-week cycles), subjects completed another 3-day food diary to assess dietary intake (T2). Subjects with a 6-week cycle did not complete the 3-day food diary at T2.

Body weight was measured before the start of the first treatment cycle, at the end of each treatment cycle and at the end of week 12. The GI tolerance questionnaire was completed on the day before the start of the first treatment cycle, at day 4 of the first treatment cycle, at the last day of the second/third treatment cycle (T2) and at the end of week 12 (T3). Quality of life was measured using the European Organisation for Research and Treatment of Cancer Quality of Life Questionnaire-Core 30 (EORTC QLQ-C30) (1, 2) and performance status, using the Eastern Cooperative Oncology Group (ECOG) score, was assessed at baseline and at the end of week 12 (3). Subjects also completed a questionnaire on changes in their taste and/or smell perception at the end of week 12. Information on treatment adherence and dose-limiting toxicities was collected by the patient's managing clinician after each treatment cycle.

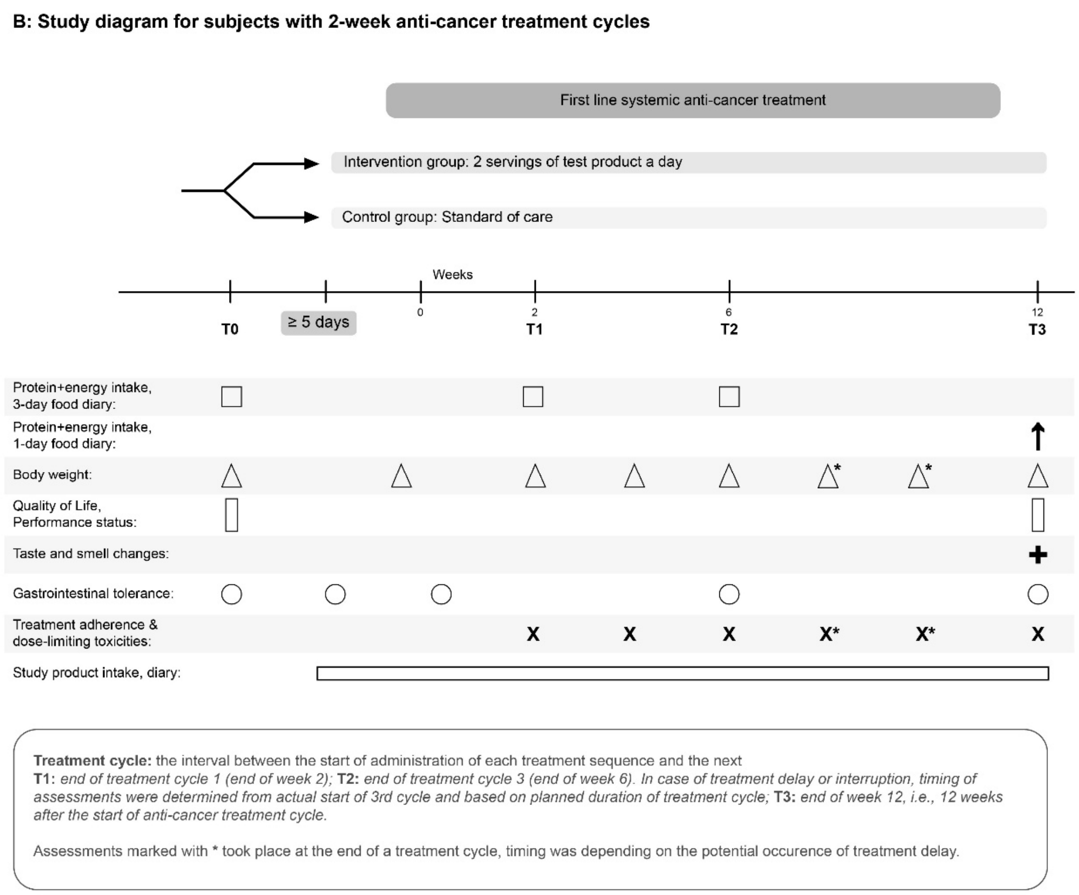

Figure S1: Schematic representation of the study design for subjects with a 2-week anti-cancer treatment cycle (B).

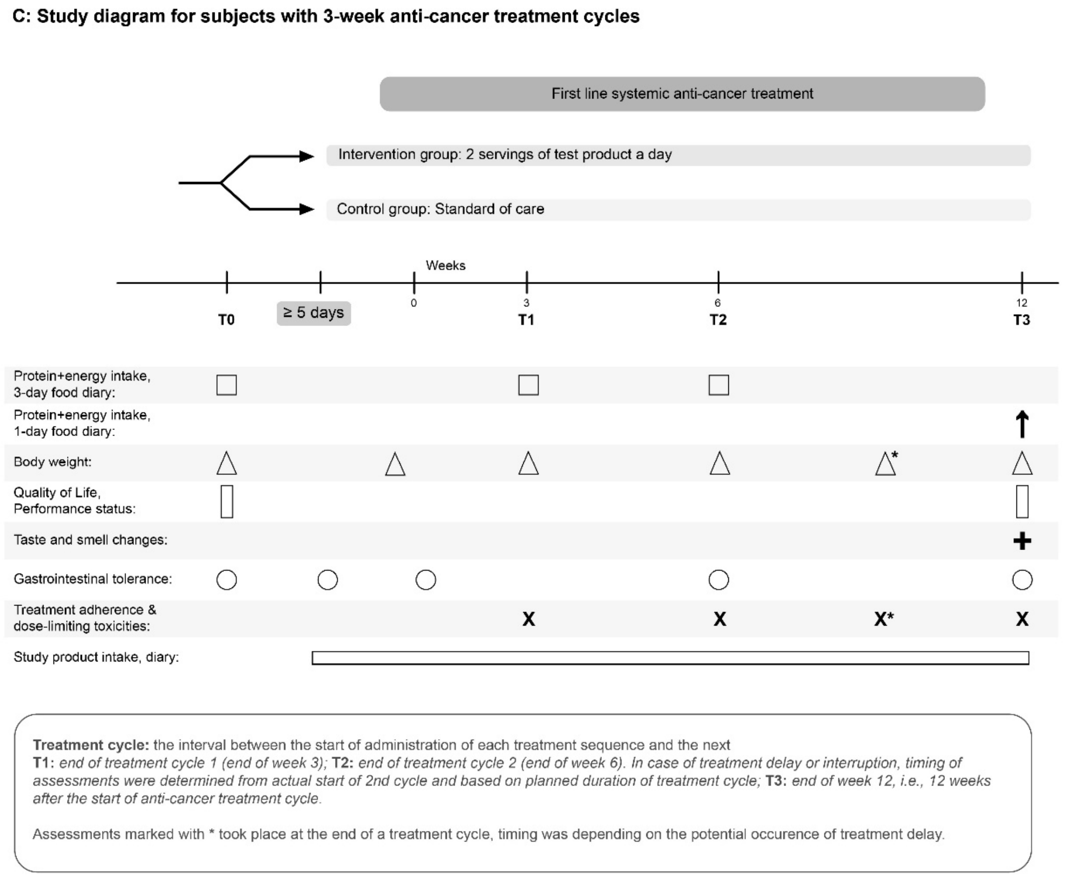

Figure S1: Schematic representation of the study design for subjects with a 3-week anti-cancer treatment cycle (C).

D: Study diagram for subjects with 6-week anti-cancer treatment cycles

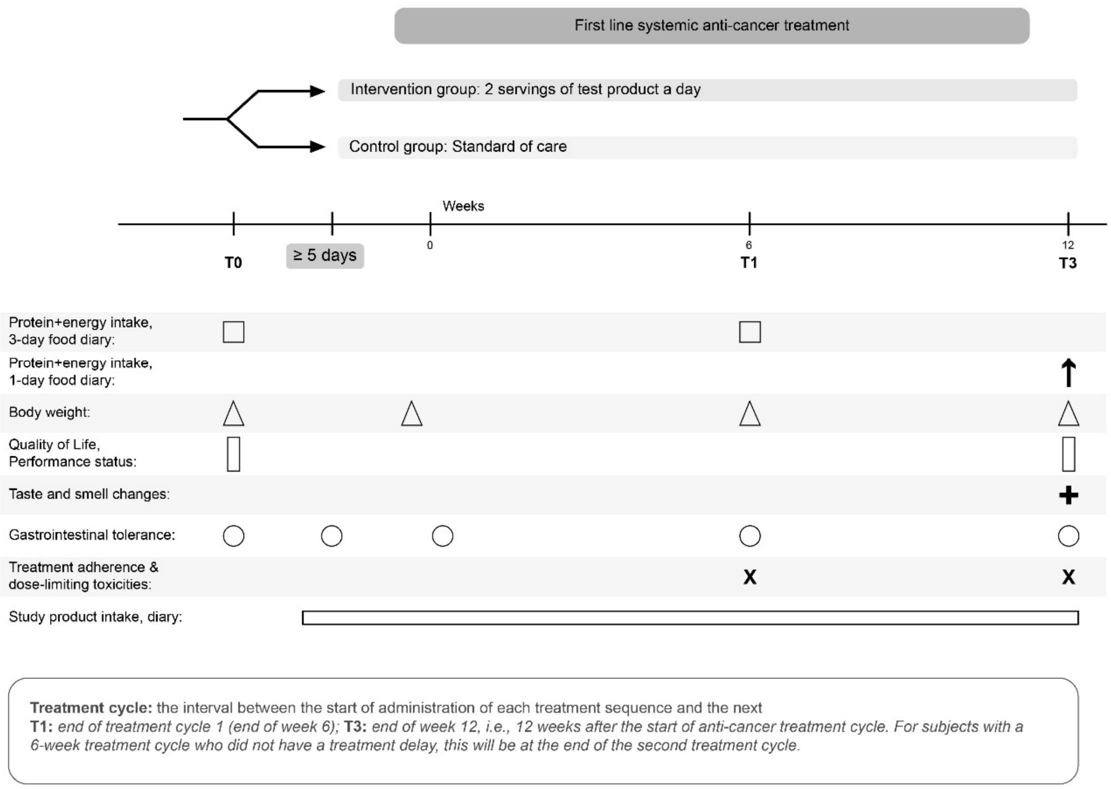

Figure S1: Schematic representation of the study design for subjects with a 6-week anti-cancer treatment cycle (D).

SUPPLEMENTARY MATERIAL

Results – additional information

Table S2: Proportion of subjects with a protein intake above the lower limit of the ESPEN recommendations (≥1.0 g/kg BW/day) for protein intake for cancer patients (mITT)

Time point	Parameter	Statistics	IG (*n*=26)	CG (*n*=11)
T0	Yes	*n* (%)	17 (65%)	5 (45%)
	No	*n* (%)	9 (35%)	6 (55%)
T1	Yes	*n* (%)	22 (88.0%)	6 (55%)
	No	*n* (%)	3 (12.0%)	5 (45%)
	Missing	*n*	1	0
T2	Yes	*n* (%)	14 (88%)	4 (40%)
	No	*n* (%)	2 (13%)	6 (60%)
	Missing	*n*	10	1
T3	Yes	*n* (%)	13 (76%)	5 (56%)
	No	*n* (%)	4 (24%)	4 (44%)
	Missing	*n*	9	2

IG, Intervention Group; CG, Control Group; BW, Body Weight; SD, Standard Deviation; mITT, Modified Intention to Treat

Table S3: Change in body weight during the 12-week study period (mITT)

Parameter	Number of subjects (IG vs. CG)	Statistics	IG (*n*=26)	CG (*n*=11)	*p*-value[1]
**Body weight (kg)**					
T0	*n*=26 vs. n=11	Mean ± SD	75.22 ± 10.49	82.34 ± 15.88	0.116
T1	*n*=26 vs. n=11	Mean ± SD	75.70 ± 10.13	82.25 ± 15.68	0.137
T2	*n*=21 vs. n=11	Mean ± SD	75.93 ± 10.42	81.87 ± 16.19	0.217
T3	*n*=18 vs. n=9	Mean ± SD	76.11 ± 10.67	84.12 ± 17.14	0.146
**Change in BW from baseline (kg)**					
T1-T0	*n*=26 vs. n=11	Mean ± SD	0.55 ± 2.47	-0.09 ± 1.77	0.449
T2-T0	*n*=21 vs. n=11	Mean ± SD	0.74 ± 2.89	-0.46 ± 2.12	0.234
T3-T0	*n*=18 vs. n=9	Mean ± SD	0.81 ± 3.92	0.84 ± 2.48	0.982

[1]*p*-value is based on *t*-test.*Statistically significant, $p \leq 0.05$. IG, Intervention Group; CG, Control Group; BW, Body Weight; SD, Standard Deviation; mITT, Modified Intention to Treat.

Results

Exploratory outcome measures – additional information

Body weight adjusted energy intake

Body weight adjusted energy intake at T2 was significantly higher in the IG compared to the CG (IG: 30.9 kcal/kg BW/day vs. CG: 23.5 kcal/kg BW/day, p=0.010 based on t-test) but not at T1 and T3.

Factors impacting dietary intake.

The majority of subjects in both groups reported no problems affecting their dietary intake at baseline (IG:77% and CG: 64%). This proportion remained stable at T1 but reduced at T2 (IG: 56% CG: 30%), before increasing again at T3 (IG: 88% CG: 80%). The most frequently reported factors were feeling full quickly, no appetite and fatigue. Changes in taste and smell perception were only experienced by a minority of the IG (taste 33%,; smell 20%,) at T3. The majority of the CG experienced a change in taste (80%) and 40% experienced a change in smell at the same timepoint.

Performance status

At baseline, fewer subjects in the IG were fully active (ECOG grade 0) compared to the CG (IG: 42%, vs. CG: 64%). At T3, performance status (ECOG grade 0) deteriorated for both groups (IG: 33% vs. CG: 22%).

Quality of life – additional information

Only gastrointestinal (GI)-related quality of life parameters such as nausea and vomiting, constipation, and diarrhoea were more present at T3 in the IG compared to the CG. Furthermore, no major differences between the IG and CG were reported for the other domains: functional scale (physical function, role function, emotional function, cognitive function, social function), symptom scale (fatigue, pain), and single items (dyspnea, sleep disturbances, appetite loss, financial impact); there were also no major differences reported between T0 and T3 within groups.

References

1. Aaronson NK, Ahmedzai S, Bergman B, Bullinger M, Cull A, Duez NJ, et al. The European Organization for Research and Treatment of Cancer QLQ-C30: a quality-of-life instrument for use in international clinical trials in oncology. *J Natl Cancer Inst.* **1993**;*85(5)*:365-76.
2. Apolone G, Filiberti A, Cifani S, Ruggiata R, Mosconi P. Evaluation of the EORTC QLQ-C30 questionnaire: a comparison with SF-36 Health Survey in a cohort of Italian long-survival cancer patients. *Ann Oncol.* **1998**;*9(5)*:549-57.
3. Oken MM, Creech RH, Tormey DC, Horton J, Davis TE, McFadden ET, et al. Toxicity and response criteria of the Eastern Cooperative Oncology Group. *Am J Clin Oncol.* **1982**;*5(6)*:649-55.
